# Age Group Differences in Response to Repeated Exposure to Laboratory Stress Tasks

**DOI:** 10.1093/geroni/igab046.2835

**Published:** 2021-12-17

**Authors:** James Miller, Gloria Luong

**Affiliations:** Colorado State University, Fort Collins, Colorado, United States

## Abstract

Research examining age differences in affect reactivity (i.e. how much affective experiences change in response to stressors) has produced mixed results, suggesting that there are areas of relative strength and weakness in regulatory processes across age-groups. The present study’s goals were to examine potential age-group differences in affect reactivity and subjective task-appraisals across repeated exposures to a psychosocial laboratory stressor. In the Health and Daily Experiences (HEADE) study, younger (18-35 years old; n=107) and older adults (60-90 years old; n=90) were exposed to the Trier Social Stress Test on three occasions in a laboratory setting over a five-day period. Current affective experiences and task-appraisals were assessed at each session using validated self-report scales, with current affective experiences measured at baseline and task periods to determine affect reactivity. Repeated measures ANOVA analyses were conducted to examine age-group differences in affect reactivity and task-appraisals across sessions. In support of our hypotheses, younger adults showed greater reductions in their negative affect reactivity over time compared to older adults [F(2, 390)= 8.18, p<.001]. Additionally, younger adults’ appraisals of task-difficulty decreased [F(2, 384)= 14.79, p<.001] and appraisals of task-performance increased [F(2,384)= 13.39, p<.001] across sessions, while older adults’ task-appraisals remained stable. Age-group differences in negative affect reactivity and task-difficulty appraisals were not evident for the first session and only emerged after repeated exposure to the stressors. These results highlight the importance of identifying age-related vulnerabilities in adapting to repeated stressors, with implications for designing effective interventions aimed at improving health and well-being for older adults.

